# Follow up after sample size re-estimation in a breast cancer trial for time to recurrence

**DOI:** 10.1186/1745-6215-14-S1-O107

**Published:** 2013-11-29

**Authors:** Erinn Hade, Gregory Young, David Jarjoura, Richard Love

**Affiliations:** 1 The Ohio State University, Columbus, OH, USA; 2 The International Breast Cancer Foundation, Madison, WI, USA; 3 University of Edinburgh, Edinburgh Clinical Trials Unit, Edinburgh, UK; 4 none, NC, USA

## 

In an international trial of premenopausal women with hormone receptor positive operable breast cancer that compares how the timing of surgical oophorectomy and mastectomy affects time to recurrence, we re-evaluated the required sample size near the end of the planned accrual period. We had anticipated that failure probabilities used at the design stage were too high resulting in a loss of power for the hazard ratio of interest. Extending follow up to obtain the number of events expected during the initial planning was not a viable option due to evidence in several previous trials of converging hazard functions. We proceeded to develop a method to re-estimate sample size in this time to event trial based on the blinded trial. Blinded re-estimation was our preference since we did not want to risk the perception that the sample size was manipulated because a smaller effect, than was anticipated, was found. Using data from a previous trial in a similar patient population receiving the same therapy (but who were not randomized to surgery timing) and the current blinded trial data, we re-estimated the required sample size. The distribution of bootstrap re-estimates of sample size indicated no increase in sample size was needed for the proposed HR. Follow-up for this trial is now complete. We will examine the final failure rates and hazard functions to see how well our re-estimation procedure performed. We will be able to explore if the hazard functions converged dramatically and what this impact would have been without this consideration.

